# ICGC PedBrain - dissecting the genomic complexity underlying medulloblastoma using whole-genome sequencing

**DOI:** 10.1186/1753-6561-6-S6-P43

**Published:** 2012-10-01

**Authors:** Natalie Jäger, David TW Jones, Marcel Kool, Thomas Zichner, Barbara Hutter, Marc Sultan, Yoon-Jae Cho, Trevor J Pugh, Volker Hovestadt, Adrian M Stütz, Tobias Rausch, Hans-Jörg Warnatz, Benedikt Brors, Paul A Northcott, Michael D Taylor, Matthew Meyerson, Scott L Pomeroy, Marie-Laure Yaspo, Jan O Korbel, Andrey Korshunov, Roland Eils, Stefan M Pfister, Peter Lichter

**Affiliations:** 1 Division of Theoretical Bioinformatics, German Cancer Research Center (DKFZ), Im Neuenheimer Feld 280, Heidelberg, 69120, Germany; 2 Division of Pediatric Neurooncology, German Cancer Research Center (DKFZ), Im Neuenheimer Feld 280, Heidelberg, 69120, Germany; 3 European Molecular Biology Laboratory (EMBL), Meyerhofstrasse 1, Heidelberg, 69117, Germany; 4 Max Planck Institute for Molecular Genetics, Ihnestrasse 63-73, Berlin, 14195, Germany; 5 Division of Child Neurology, Stanford University, 750 Welch Road, Palo Alto, CA 94304, USA; 6 Broad Institute of MIT and Harvard, Cambridge, MA 02142, USA; 7 Division of MolecularGenetics, German Cancer Research Center (DKFZ), Im Neuenheimer Feld 280, Heidelberg, 69120, Germany; 8 Division of Neurosurgery and The Arthur and Sonia Labatt Brain Tumour Research Centre, Hospital for Sick Children, 555 University Avenue, Toronto, Ontario, Canada, M5G 1X8; 9 Dana Farber Cancer Institute, 450 Brookline Avenue, Boston, MA 02215, USA; 10 Children's Hospital Boston, 300 Longwood Avenue, Boston, MA 02115, USA; 11 Department of Neuropathology, University of Heidelberg, Im Neuenheimer Feld 220, Heidelberg, 69120, Germany; 12 Clinical Cooperation Unit Neuropathology, German Cancer Research Center (DKFZ), Im Neuenheimer Feld 280, Heidelberg, 69120, Germany; 13 Institute of Pharmacy and Molecular Biotechnology, and Bioguant Center, University of Heidelberg, Im Neuenheimer Feld 267, Heidelberg, 69120, Germany; 14 Department of Pediatric Oncology, Hematology & Immunology, Heidelberg University Hospital, Im Neuenheimer Feld 430, Heidelberg, 69120, Germany

## Background

Medulloblastoma is the most common malignant brain tumor in childhood. It arises in the cerebellum or medulla/brainstem, and shows tremendous biological and clinical heterogeneity. Despite advances in treatment for medulloblastoma over the past few decades, approximately 40% of children who develop this aggressively growing malignancy will experience tumor recurrence, and 30% will die from the disease. Importantly, recent work has shown that medulloblastoma is not a single disease, but comprises at least four distinct molecular subgroups [1]. WNT tumors, displaying activated wingless pathway signaling, carry a favorable prognosis. SHH tumors show hedgehog pathway activation, and have an intermediate prognosis. Group 3 and 4 tumors are molecularly less well characterized, and present the greatest clinical challenges. The full repertoire of genetic events driving this distinction, however, remains unclear.

We have recently described an integrative deep-sequencing analysis of 125 tumor-normal pairs, conducted as part of the International Cancer Genome Consortium (ICGC) PedBrain Tumor Project [2]. Here, we focus on genome-wide somatic mutations in medulloblastoma, how they are distributed throughout the genome, how they are correlated with patient age at diagnosis, the influence of subgroup affiliation on mutation rate, and how the mutation allele frequencies can be utilized to predict ploidy and infer temporal evolution of the tumor.

## Materials and methods

We sequenced the complete genomes of 39 primary medulloblastoma and matched normal DNAs from the same individuals, aged from 0 to 17 years, using Illumina technology. Cancer and normal DNAs were sequenced to an average of 35-fold coverage and analyzed to identify somatic base substitutions, small insertions, deletions, and copy number changes.

## Results

Tetraploidy was identified as a frequent event in clinically challenging group 3 and 4 tumors. The extremely low fraction of mutations at approximately 50% allele frequency indicates that genome duplication occurred very early during tumorigenesis. For non-tetraploid tumors, a clear positive correlation of patients' age and mutation number was observed. The average somatic mutation rate was 0.52 per megabase (Mb), with an average of 10.3 non-synonymous coding single nucleotide variants, amounts considerably lower than in deep-sequenced adult malignancies. SHH tumors harboring *TP53* mutations showed a significantly higher mutation rate both genome wide and for non-synonymous changes. Interestingly, the WNT subgroup, which typically shows a good prognosis, had the next highest mutation rate. Several recurrent coding mutations were identified, both in known medulloblastoma-related genes and in genes not previously linked to this tumor, often in subgroup-specific patterns. Chromatin modifiers were frequently altered across all subgroups.

## Conclusions

Next-generation sequencing of this large cohort has provided a detailed insight into new mechanisms contributing to medulloblastoma tumorigenesis, enhancing our understanding of the genomic complexity and heterogeneity underlying medulloblastoma, and providing several potential targets for new therapeutics.
